# Interactions between intrinsic and extrinsic factors shape island bat survival and extirpation in a global extinction hotspot

**DOI:** 10.1093/evolinnean/kzag014

**Published:** 2026-06-15

**Authors:** Kristen M Rayfield, Siobhán B Cooke, Alexis M Mychajliw, Samuel T Turvey, Nathan Upham, Sarah Vaccaro, Liliana M Dávalos

**Affiliations:** Department of Ecology and Evolution, Stony Brook University, Stony Brook, NY 11794, United States; Center for Functional Anatomy and Evolution, Johns Hopkins University School of Medicine, Baltimore, MD 21205, United States; Department of Biology, Middlebury College, Middlebury, VT 05753, United States; Program in Environmental Studies, Middlebury College, Middlebury, VT 05753, United States; Institute of Zoology, Zoological Society of London, London, NW1 4RY, United Kingdom; School of Life Sciences, Arizona State University, Tempe, AZ 85281, United States; Department of Ecology and Evolution, Stony Brook University, Stony Brook, NY 11794, United States; Department of Ecology and Evolution, Stony Brook University, Stony Brook, NY 11794, United States; Institute for Advanced Computation Science (IACS), Stony Brook University, Stony Brook, NY 11794, United States; Consortium for Inter-Disciplinary Environmental Research (CIDER), Stony Brook University, Stony Brook, NY 11794, United States

**Keywords:** body mass, extirpation, Antillean, Bayesian hierarchical modelling

## Abstract

Caribbean islands are hotspots of mammalian biodiversity and extinction where only a few intermediate-sized non-volant species survived. Despite numerous extirpations and extinctions, how body size (intrinsic trait) or island features (extrinsic environmental variables) contribute to the survival and extirpation of 479 Caribbean bat populations is poorly known. We used phylogenetic hierarchical models to impute missing body mass data for several fossil species and then to model extirpation probability as a function of intrinsic traits and extrinsic variables. We found smaller- and larger-sized bat species had higher rates of survival than intermediate-sized ones; elevation increased, island size decreased this curvilinear survival probability, and deforestation decreased overall survival. We propose that: (i) flight constrains body size such that bats are prey to introduced species but larger bats can better recolonize islands, yielding a pattern of greater survival at larger sizes, (ii) undersampling of bat populations on small islands and the diversity of introduced species on larger islands explains the interaction with island size, and (iii) deforestation reflects centuries of long-standing biotic reconstruction that transformed island landscapes. Our findings highlight the primacy of body mass and its interaction with extrinsic variables in explaining Caribbean bat extirpations with broader implications for island biogeography, evolution, and conservation.

## Introduction

Islands make up less than 8% of the global land area, yet they are hotspots for biodiversity and harbour ∼20% of the world’s biota (Kier *et al.* 2009, Jones *et al.* 2010). Factors such as area and isolation further shape their biodiversity and extinction patterns (Koopman 1958, Gillespie *et al.* 2012), with larger islands typically supporting populations that are larger and more resilient to extinction. In a quantitative synthesis, the equilibrium theory of island biogeography posits that island species richness reaches an equilibrium between rates of colonization —a function of geographic isolation—, and extinction, a function of island size (MacArthur and Wilson 1967). Islands are, however, dynamic systems that change in size and isolation through geological or climatic processes (Whittaker *et al.* 2017), and recent studies show they are often in biogeographic disequilibrium (Heaney 2000, Valente *et al.* 2015, 2017). This challenges long-held assumptions about island characteristics and species traits that influence resilience and extinction risk. Because of their limited geographic area, island ecosystems are particularly vulnerable to climate-related weather events such as hurricanes, and anthropogenic changes including invasive species introduction and habitat fragmentation and loss (Jones *et al.* 2010). However, population responses to such changes and their resilience or eventual extinction also hinge on intrinsic traits that mediate persistence under changing biotic and abiotic conditions.

Bats (Chiroptera) are an ideal clade with which to test macroecological hypotheses of extinction selectivity. Bats are among the most taxonomically diverse and geographically widely-distributed mammals and with more than 1500 described species, they comprise ∼20% of extant mammalian taxa (Simmons and Cirranello 2025). Nearly 60% of bat species inhabit islands, and play critical roles in maintaining ecosystem structure and function through seed dispersal, pollination, and regulating insect populations (Jones *et al.* 2010, Gallant *et al.* 2020, Ferreira *et al.* 2022). Among mammals, their exceptional dispersal ability has allowed them to successfully colonize multiple island systems (Jones *et al.* 2010), making them suitable for analysing population extirpation risk among the same species on different islands that vary in abiotic and biotic factors.

Here, we leverage the three distinct Caribbean island systems (Bahamas “Lucayan Archipelago”, Greater Antilles, Lesser Antilles) to explore the interplay of trait, environmental, and biogeographic variables in shaping extinction dynamics and biodiversity patterns among insular bat populations. We further use these island systems as a series of replicates to assess how similar conditions across multiple islands may yield distinct colonization and extinction patterns (Ricklefs and Bermingham 2008). Geographically, the Caribbean comprises the Lucayan or Bahamian Archipelago, the Greater Antilles, and the Lesser Antilles. The low-lying islands of the Lucayan Archipelago are composed of marine carbonate sediments scattered on shallow banks, whereas the Greater Antilles are remnants of continental crust with high mountain ranges and karst landscapes, and at the eastern margin of the Caribbean plate, the Lesser Antilles form an arc of geologically active volcanoes (Ricklefs and Bermingham 2008, Mohammed *et al.* 2022). Within each of these three regions, Caribbean islands have diverse sizes, geomorphic forms, geotectonic histories, and ecological trajectories shaped by local historical contingencies and human alterations (Kemp *et al.* 2020a, b).

In alignment with their complex biogeographic features, the Caribbean islands supported a diverse Quaternary terrestrial mammal fauna that experienced more extinctions than any other region since deglaciation at the Pleistocene–Holocene transition (Turvey 2009, Dávalos and Turvey 2012, Cooke *et al.* 2017, Orihuela *et al.* 2020). Of the > 150 Quaternary endemic mammalian species (51 of these bats), only 13 non-volant mammalian species and 41 bat species survive today, with a loss of roughly 90% of non-volant mammals and only ∼19% of bat species. (Dávalos and Turvey 2012, Cooke *et al.* 2017). From the mid-Holocene onwards, the arrival of different waves of human colonists has been associated with varying patterns of mammal extinction events (Fitzpatrick and Keegan 2007, Dávalos and Turvey 2012, Cooke *et al.* 2017, LeFebvre *et al.* 2019, Turvey *et al.* 2021). These events were driven by direct overexploitation, habitat conversion, and landscape modification, and the introduction of invasive species and subsequent predation, competition, and enzootic diseases, and had severe impacts on non-volant mammals (Cooke *et al.* 2017, Orihuela *et al.* 2020). Both the nature and ecological consequences of these stressors varied across the Caribbean, reflecting differences in island size, biotas, and colonial land use (Hooghiemstra *et al.* 2018). Contemporary Caribbean island landscapes thus represent a palimpsest of biotas reconstructed over time.

Body mass is an integrative proxy for multiple functional attributes that collectively shape species’ ecological roles and that drive the structure and functioning of communities. It is recognized as a major determinant of extinction risk in mammals (Cardillo 2003, Cardillo *et al.* 2005, Matthews *et al.* 2011, Ripple *et al.* 2017). Among terrestrial mammals, larger-bodied species are generally more at risk of extinction due to their lower population densities and slower growth rates, thus making them more susceptible to environmental disturbances and human exploitation (e.g. hunting) (Cardillo *et al.* 2005). However, global analyses of extinction risk on island systems indicate that both small-bodied and large-bodied species can be more vulnerable than those of intermediate size (Lyons *et al.* 2016, Rozzi *et al.* 2023). The disproportionate survival of intermediate-sized species across the Caribbean non-volant mammal fauna has been called the ‘Goldilocks Hypothesis’ (Hansford *et al.* 2012). In the Caribbean system, intermediate-sized species are hypothesized to have been at reduced risk of direct human exploitation but were large enough to withstand invasive predators, thus making their size ‘just right’ and resilient to extinction (Turvey *et al.* 2021). However, the relationship between body mass and extinction risk in bats in the Caribbean remains to be explored. Because extinction usually arises through interactions between species traits and spatially variable environmental change, here, we examine both intrinsic and extrinsic factors for the first time to quantitatively model all known bat island-level extirpation events in the Caribbean.

## Material and methods

### Data collection

#### Geographic distributions and taxonomy

**Figure 1:**
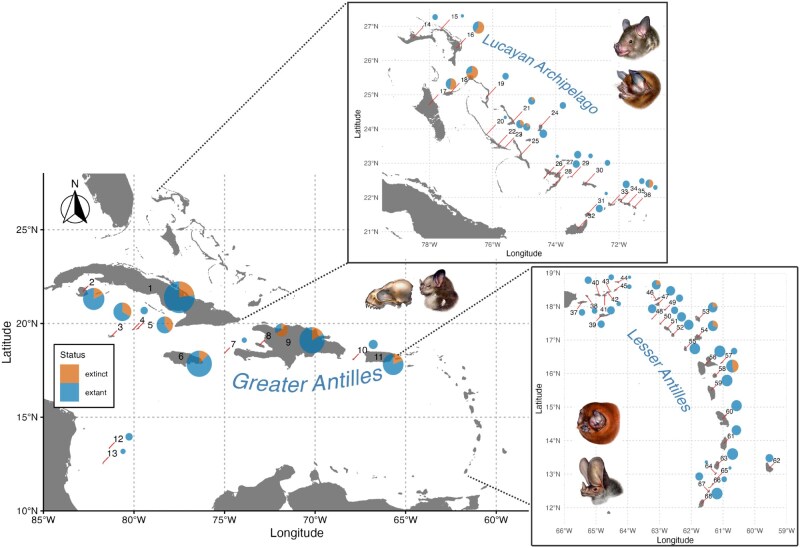
Map of the Caribbean Islands. The maps were generated in R (v.4.4.1) using the rnaturalearth package (v.1.2) (Massicotte and South 2026) and the geodata package (v.0.6.2) (Hijmans *et al*. 2021) to download country specific data from the GADM database (v.4.1) for the Bahamas (GADM 2022). Numbers refer to islands, the scale of the pies indicates the total number of bat species (extinct and extant) on the islands, with illustrations of some extinct or extirpated bat species. Main map: 1. Cuba; 2. Juventud; 3. Grand Cayman; 4. Little Cayman; 5. Cayman Brac; 6. Jamaica; 7. Navassa; 8. Gonave; 9. Hispaniola; 10. Mona; 11. Puerto Rico; 12. Providencia; 13. San Andres. Top left: skull illustration of extinct *Cubanycteris silvai*. Top right: illustration of *Phyllops falcatus*. Lucayan Archipelago Map: 14. Grand Bahama; 15. Little Abaco; 16. Great Abaco; 17. Andros; 18. New Providence; 19. Eleuthera; 20. Darby; 21. Cat Island; 22. Great Exuma; 23. Little Exuma; 24. San Salvador; 25. Long Island; 26. Fortune Island; 27. Crooked Island; 28. Acklins; 29. East Plana Cay; 30. Mayaguana; 31. Little Inagua; 32. Great Inagua; 33. Providenciales; 34. North Caicos; 35. Middle Caicos; 36. East Caicos. Top right: illustration of *Brachyphylla nana*. Bottom right: illustration of *Pteronotus parnellii*. Lesser Antilles Map: 37. Vieques; 38. Culebra; 39. Saint Croix; 40. Saint Thomas; 41. Saint John; 42. Tortola; 43. Guana; 44. Anegada; 45. Virgin Gorda; 46. Anguilla; 47. Saint Martin; 48. Saba; 49. Saint Barthelemy; 50. Saint Eustatius; 51. Saint Kitts; 52. Nevis; 53. Barbuda; 54. Antigua; 55. Montserrat; 56. Guadeloupe; 57. La Desirade; 58. Marie Galante; 59. Dominica; 60. Martinique; 61. Saint Lucia; 62. Barbados; 63. Saint Vincent; 64. Bequia; 65. Mustique; 66. Union; 67. Carriacou; 68. Grenada. Middle left: illustration of *Mormoops blainvillei*. Bottom left: illustration of *Macrotus waterhousii*. Illustrations by Adrián Tejedor.

We compiled a dataset comprising 64 extant and 12 extinct bat species (67% endemic to the Caribbean Islands) from six families (Molossidae, Mormoopidae, Natalidae, Noctilionidae, Phyllostomidae, and Vespertilionidae) across 68 Caribbean islands (Fig. 1). This dataset builds on that of Morgan (2001) and Dávalos and Turvey (2012), with the following major changes: *Mormoops megalophylla* is not endemic to the Caribbean islands, *Phyllonycteris major* is present in the Quaternary fossil record on Antigua (Steadman *et al.* 1984), and *Natalus primus* was rediscovered as living on Cuba (Tejedor *et al.* 2004) (details on minor changes to the dataset are in the corresponding script). This dataset was further updated to include *Artibeus lituratus* and *Artibeus schwartzi* in the fauna of Carriacou (Genoways *et al.* 2010); remove *Artibeus schwartzi* and replace *Myotis martiniquensis* with *Myotis nyctor* on Barbados; assign *Artibeus schwartzi* to St. Lucia; remove *Artibeus jamaicensis* and *Artibeus planirostris* and include *Micronycteris buriri* in the fauna of St. Vincent (Genoways *et al.* 2012); update the taxonomy of *Sturnira* so that the former Antillean ‘*lilium*’ is referred to *paulsoni*, and *angeli* includes the former ‘*thomasi*’ (Velazco and Patterson 2013); include *Monophyllus plethodon* as extant and include *Mormoops blainvillei*, *Mormoops megalophylla*, *Pteronotus parnellii*, *Pteronotus macleayii*, *Pteronotus quadridens*, *Phyllonycteris major*, *Natalus major*, and *Tadarida brasiliensis* as extinct on Marie Galante (Stoetzel *et al.* 2016); remove *Tadarida* sp. as an extinct species, add *Pteronotus macleayii* and *Lasiurus cinereus* to the extant fauna of Hispaniola (Soto-Centeno *et al.* 2017) and elevate *Lasiurus insularis* to species level and subsume *Lasiurus intermedius* (Simmons and Cirranello 2025). To align with Turvey *et al.* (2021), we include Grenada despite its bat fauna being continental South American instead of insular (Koopman 1958, Genoways *et al.* 2010).

#### Traits

We compiled data on bat body mass, dietary patterns, and roosting patterns. For extant taxa, body mass data were collected using published direct measurements and field measurements (Supplementary Table S1). A complete list of body mass data and sources is presented in Supplementary Table S2. For non-flying mammals, published regression equations are often used to infer body mass of unavailable taxa (e.g. Turvey *et al.* 2021), but those for bats (e.g. Giannini *et al.* 2012) rely on skeletal measurements that are unavailable for many fossil species. Therefore, to infer the body mass of 12 species often extinct on the islands and without continental representatives, we compiled a dataset of greatest length of skull (GLS) for the entire sample. We used published measurements and estimates of the mean, combining data for males and females (Supplementary Table S3). However, the extinct species *Mormoops magna* has only been identified through humeral fragments, and together with *Pteronotus sp. nov.* of Morgan (2001), lacked GLS (expanded under statistical analyses below). We compiled data for roosting and dietary traits using published records. We separated classified species into 0 for non-cave roosters and 1 for those using obligate cave roosts (Supplementary Table S4), and classified diet into 0 for insectivores and 1 for omnivores and herbivores (Supplementary Table S5).

#### Island environmental characteristics

We updated the data on Caribbean island characteristics published by Turvey *et al.* (2021), to include comparative data for 22 additional islands also occupied by bats but not non-volant mammals. We calculated the area, maximum and mean elevation, proportion of forest cover in 2000 and loss in 2014, and mean Human Footprint Index (a global dataset that measures human influence such as land use, roads, access on terrestrial environments), as outlined in Turvey *et al.* (2021), although not all values were obtained for all islands (Supplementary Table S6). We also incorporated bathymetry-based estimates of the Last Glacial Maximum (LGM) island areas (Supplementary Table S7). These areas were then used to compute a new area loss variable for subsequent use as a covariate.

#### Phylogenies

To obtain phylogenies for analyses, we randomly sampled 100 phylogenies from http://vertlife.org/phylosubsets/ (Upham *et al.* 2019). Following Turvey *et al.* (2021), we grafted missing species (*N* = 16) to these phylogenies using their taxonomy in the *bind.tip* routine in the R package phytools (Revell 2024), and then arbitrarily resolved polytomies using multi2di in the R package ape (Paradis and Schliep 2019). To meet Bayesian model assumptions, short branch lengths of 0.0001 were added to the resolved polytomies of the grafted internodes. We tested the robustness of our results to a different mammalian phylogeny by rerunning these analyses with mean branch lengths tree from Álvarez-Carretero *et al.* (2022).

### Statistical analyses

Analyses were conducted in R v.4.4.2 (R Core Team 2024). We applied a hierarchical Bayesian approach to both impute missing trait values for a subset of species and estimate coefficients for species traits and island covariates. To avoid confusion in the types of effects included in regressions, we use the terms ‘group-specific’ instead of ‘random’ and ‘sample-wide’ instead of ‘fixed’ (Gelman 2005).

#### Inferring missing trait measurements using Bayesian phylogenetic models

We implemented Bayesian phylogenetic regressions to impute missing trait values for several species. We implemented the general approach outlined by Gómez-Rubio (2020) using the integrated nested Laplacian approximations implemented in the INLA R package (Rue *et al.* 2016), while also using the phyr R package for some steps (Li *et al.* 2020). Twelve species were missing body mass estimates: of these, *Sturnira paulsoni*, *Lasiurus degelidus*, and *Lasiurus minor* were extant and the remainder were extinct. Two species, *Mormoops magna* and *Pteronotus sp. nov.*, lacked data for both GLS and body mass. To impute the GLS value, we first modelled log_10_-transformed GLS as a function of log_10_-transformed body mass using the Phylogenetic Generalized Linear Mixed Model for Community Data *pglmm* routine in phyr, with a single phylogeny to estimate cluster-specific effects that co-vary phylogenetically and bayes = T to apply INLA. As 12 species in the complete dataset lacked body mass data, those species were not used to estimate the GLS coefficient. We then drew 100 samples from the posterior distribution of the GLS imputation model using the *inla.posterior.sample* routine in the INLA package. These samples included imputed GLS values. We then modelled log_10_-transformed body mass as a function of log_10_-transformed GLS in a loop of 100 *inla* iterations in INLA such that each of the 100 imputed GLS values were included in the data. This enabled us to compile a frequency distribution of imputed body mass values for each of the 12 species missing body mass and thus include a probabilistic range of imputed values in inferring extirpation probability downstream. We summarized these imputation models using the *inla.merge* routine in INLA. We repeated this procedure with the phylogeny of Álvarez-Carretero *et al.* (2022). Assuming a normal data distribution, we categorized body size into small (<8.6 g), intermediate (9–22 g), and large (>23 g) using quantiles.

#### Modelling extirpation as a function of intrinsic traits and extrinsic island environments

Because previous work on extinction risk for Caribbean non-volant mammals supported greater probability of survival for intermediate-sized species, we also include this option here, though we note that all Caribbean bats fall within the range of ‘small mammals’ when including volant and non-volant Caribbean mammals (Turvey *et al.* 2021). To do so, we log_10_-transformed, scaled, centred, and squared body mass values, making body mass^2^ values positive and including these as a covariate. Environmental variables may covary, resulting in multicollinearity and unstable covariate coefficients. We tested for collinearity, excluding one of any pair of variables with a significant correlation coefficient ≥ 0.70, as Bayesian regressions are robust to imperfectly collinear predictors (Pesaran and Smith 2019).

To make coefficients comparable across covariates, environmental variables were transformed as summarized in Table 1. Based on previous results supporting an interaction between non-volant mammals’ body mass and human arrival, we also evaluated interactions between body mass and squared body mass and environmental characteristics previously implicated in mammalian survival or extinction, such as area or elevation (Dávalos and Russell 2012, Turvey *et al.* 2021).

**Table 1:** Covariates in extirpation model, level of observation, and transformations.

Covariate	Factors	Level	Transformation
Body mass	Intrinsic	Species	Log_10_ and scale
Body mass^2^	Intrinsic	Species	${\mathrm{mass}}_{\mathrm{transformed}}^{2}$
Cave	Intrinsic	Species	None, binary
Diet	Intrinsic	Species	None, binary
Area	Extrinsic	Island	Log_10_ and scale
Area change (since LGM)	Extrinsic	Island	Log_10_ $\frac{{\mathrm{area}}_{\mathrm{current}}}{{\mathrm{area}}_{\mathrm{last}\;\mathrm{glacial}\;\mathrm{maximum}}}$
Elevation	Extrinsic	Island	+1, log_10_ and scale
Hurricane frequency	Extrinsic	Island	Scale
Volcano	Extrinsic	Island	None, binary
Proportion forest cover 2000	Extrinsic	Island	Scale
Proportion forest loss to 2000	Extrinsic	Island	+1, log_10_ and scale
Mean human footprint index (to 2005)	Extrinsic	Island	Scale
First human arrival	Extrinsic	Island	Log_10_ and scale

In line with previous work (Turvey *et al.* 2021), each observation corresponded to a species-island combination. Each observation was modelled as a single-trial binomial response of the probability of survival for that species on the island, *pr_i_*, distributed such that:


$$y_{i}\;∼\mathrm{dbern}({pr}_{i})$$

$${\mathrm{logit}({pr}_{i})=\beta_{0}+\beta_{1}X}_{i}+b_{0}+b_{\mathrm{species}}+b_{\mathrm{islands}}$$

With sample-wide effects for the global intercept $\beta_{0}$ and the suite of coefficients on covariates by species or islands represented by $\beta_{1}$. Non-phylogenetic species-specific intercepts are given by:


$$b_{0}\;∼\mathrm{Gaussian}(0,\;\sigma_{0}^{2}I_{\mathrm{species}})$$

The phylogenetic variance–covariance $V_{\mathrm{species}}$ also generates species-specific effects modelled by:


$$b_{\mathrm{species}}\;∼\mathrm{Gaussian}(0,\;\sigma_{0}^{2}V_{\mathrm{species}})$$

And uncorrelated island-specific effects are modelled by independent intercepts given by:


$$b_{\mathrm{islands}}\;∼\mathrm{Gaussian}(0,\;\sigma_{0}^{2}I_{\mathrm{islands}})$$

These models were implemented using the *pglmm* routine in phyr with a complexity penalizing prior (Simpson *et al.* 2017), and repeated for the phylogeny of Álvarez-Carretero *et al.* (2022). We used the *simulateResiduals* routine in the DHARMa R package (Hartig 2022) to investigate how the data fit model assumptions. To calculate model *R^2^*, we used the *R^2^* routing in the R package rr2 (Ives 2019). As this was a binomial regression, we used several routines in the R package pROC (Robin *et al.* 2011) to calculate model sensitivity, specificity, and area under the receiver operating characteristic (ROC) curve (AUC) (Allouche *et al.* 2006). An AUC of 0.5 is considered random, with higher values up to 1 indicating a progressively better model.

To account for variance across both estimates of body mass and phylogenies, we iterated this model 100 times varying the trait estimate and phylogeny from the sample by Upham *et al.* (2019) in each case. We then used custom scripts to extract summaries (i.e. median and 2.5% to 97.5% of the posterior marginals) of the posterior estimates of coefficients from each model iteration. To plot the sample-wide coefficients we used the *Efxplot* routine of the R package ggregplot (Albery 2019).

#### Species-area and species-species relationships

Island size is a determinant of the number of species (MacArthur and Wilson 1963). To examine how island size influenced the number of bat species in the Caribbean, we compiled the numbers of total bat species, extinct bat species, and introduced (all non-volant) species (Kemp *et al.* 2020a, b) per island (Supplementary Table S8). We then built linear models of species as a function of island size, with both values in log_10_ scale. We also modelled the number of extinct bat species as a function of the total number of bat species. In addition, we collected last occurrence dates for extinct bat species that had radiocarbon dates as well as earliest human arrival dates to islands where bat extinctions occurred (Supplementary Table S9).

## Results

Our analyses revealed many more island population extirpation events than extinction events among 76 Caribbean bat species. In contrast with patterns of endemism among non-volant mammals (Turvey *et al.* 2021), most bat species had populations on more than one island. This pattern persisted across island groups with differing LGM island connectivity. Extirpated populations comprised, on average, smaller-bodied species than extant ones (Table 2). A contingency analysis of survival vs. single- or multi-island range (Table 2) was significant ($X_{1}^{2}$ = 13.598, *P*value < 0.0002); more single-island species go extinct.

**Table 2:** Island population distribution across islands for extant and extinct or extirpated Caribbean bat species. For extinct bat last occurrence dates, see Supplementary Table S9.

Island population description	Putative extant LGM	Extant Holocene	Putative extinct LGM	Extinct Holocene
Number of species	64^a^	64	1	12
Number of island populations	270^a^	401	69^a^	78
Species populations living on only one island	26^a^	24	17^a^	15
Species populations present on more than one island	244^a^	377	52^a^	63
Mean body mass (g) of populations (log_10_)		1.223 ± 0.017		1.067 ± 0.032

a The last occurrence dates for many island populations are unknown and so their status during the Last Glacial Maximum (LGM) is unknown.

### Missing trait measurement inference

The phylogenetic model of GLS as a function of body mass described an equation:


$$\log_{10}GLS\;=\;0.985+0.260*\log_{10}m\mathrm{ass}$$

In addition to significant phylogenetic effects, this model was predictive with a marginal (sample-wide) *R^2^* of 0.27 and a conditional (all effects) *R^2^* of 0.84. Random draws from the distribution of this model estimate the GLS of *Mormoops magna* with a mean of 15.8 mm and SD of 1.2 mm (alternate phylogeny: mean of 13.66 mm, SD of 1.2 mm) and that of *Pteronotus sp. nov.* at a mean of 17.2 mm and SD of 1.2 mm (alternate phylogeny: mean of 16.64 mm, SD of 1.2 mm). Likewise, the body mass-to-GLS model is described by:


$$\log_{10}m\mathrm{ass}\;=\;-2.162+2.544*\log_{10}GLS$$

For a less predictive model with a marginal *R^2^* of 0.15 and a conditional *R^2^* of 0.84. Body mass estimates for the 12 species lacking observations and based on both the Upham *et al.* (2019) and Álvarez-Carretero *et al.* (2022) phylogenies are summarized in Supplementary Table S10.

### Intrinsic and extrinsic covariates of Caribbean bat extirpation and survival

Significant positive correlation (*R *= 0.94, *P *= 6.03E-33) was detected between average elevation and maximum elevation in the island dataset; to make these analyses comparable to previously published ones, only average elevation was subsequently analysed (Supplementary Fig. S1). Combining the bat trait and geographic distribution data yielded 479 observations of extirpation or survival of bat species by island. Of these, 455 rows had non-imputed body mass observations.

We began by including all potential covariates of extinction. However, wide intervals for the coefficient of volcano prompted cross-tabulation with the response. We found all of the 78 extirpation events were recorded on islands lacking an active Holocene volcano, while the remaining observations of survival were distributed between islands with and without volcanoes. This quasi separation yields unstable coefficients (Albert and Anderson 1984), so the volcano covariate was dropped from subsequent analyses. We also tested for phylogenetic attraction (whether the phylogenetic covariance of species might explain island faunas), but this nested effect was not significant (likelihood ratio test, *LRT*_1_ = 0, *P *= 1).

Three initial analyses with a single phylogeny explored both the impact of body mass imputation and the inclusion of different covariates. First, we analysed the dataset without body mass imputation (*N *= 455 rows) with all covariates. Second, the body mass imputation dataset (*N *= 479 rows) with all covariates. Last, we included only statistically supported covariates (*N *= 479 rows). Results were consistent across all three models as well as to the alternative phylogeny (Supplementary Table S11), and we subsequently report results from the second analysis including imputed body mass data. Residual plots showed no evidence of strong model violations (Supplementary Fig. S2). The resulting model had a *R^2^* of 0.68, with high sensitivity (0.86) and higher specificity (0.97), and a very high AUC of 0.977.

**Figure 2:**
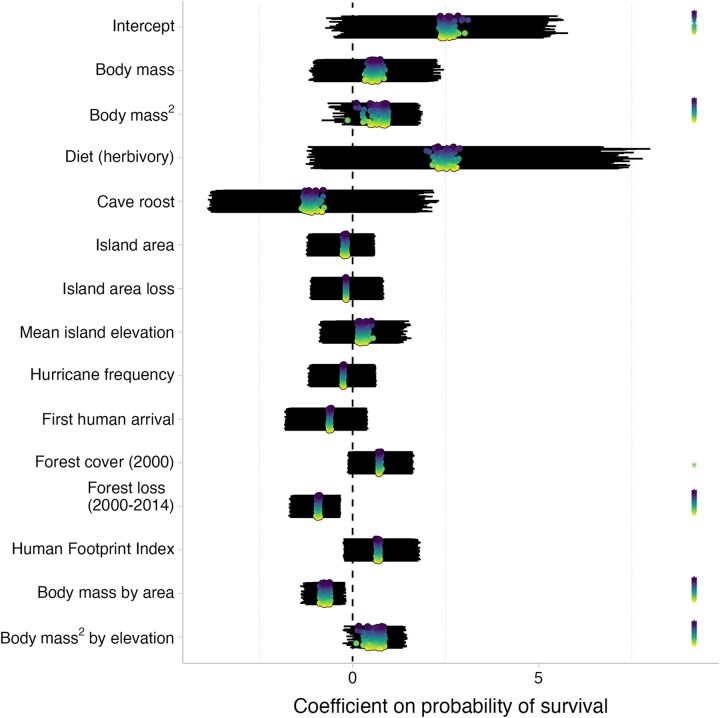
Results from analyses of per-island bat survival probability in the Caribbean Islands modelled as a function of intrinsic and extrinsic variables. Coefficient estimates for the sample-wide effects of models accounting for variation in both phylogeny and imputed body mass are on the x axis. Each colour dot represents the mean coefficient estimate, with the line encompassing the 95% credible interval (CI) across these models, for one of 100 models that randomly sampled mammalian phylogenies and posterior estimates of body mass for species in Supplementary Table S10. Asterisks indicate the CI does not overlap with 0 and the coefficient estimates are statistically supported.

Posterior estimates accounting for variation in both the phylogeny and body mass imputation were consistent with the single-phylogeny analyses (Fig. 2). The intercept of the regression was positive and often exceeded zero, indicating that most bat species on individual islands survived. While body mass was a positive covariate, its effect overlapped with zero. In contrast the effect of body mass^2^ was positive and statistically supported, indicating non-linear effects of body mass on the probability of extirpation. No other intrinsic variables were statistically supported covariates. Third, among the extrinsic island covariates, forest loss reduced probability of survival. Last, body mass interacted with both island size and elevation, yielding a negative coefficient on survival for the first and positive one for the second. Non-phylogenetic species effects (Supplementary Fig. S3) and island-specific effects (Supplementary Fig. S4) only showed an effect for Cayman Brac, with lower probability of survival.

**Figure 3:**
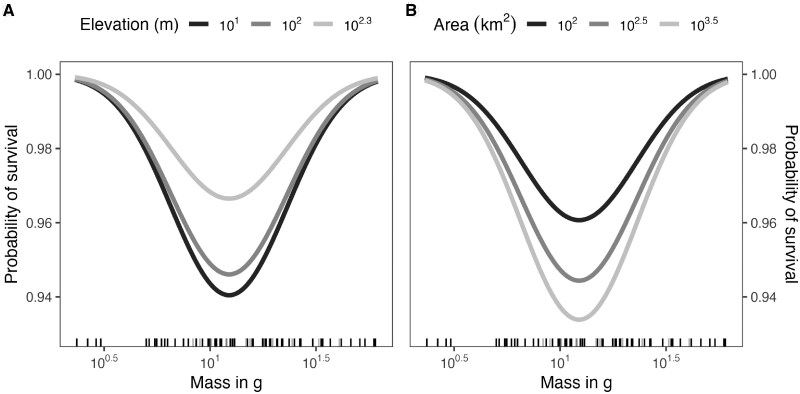
Nonlinear influence of body mass on the probability of survival of Caribbean island bat species on y axis with body mass on the x axis. Rugs show observed (black) and inferred (grey) masses. A, body mass by island elevation interaction. B, body mass by island area interaction.

To better interpret the sample-wide results, Figure 3 summarizes the effect of body mass on the probability of survival across three levels of island area (Fig. 3A) and elevation (Fig. 3B). Smaller islands slightly increased bat survival probability, while slightly lower-elevation islands decreased bat survival probability. Strikingly, bat species at intermediate body sizes within the spectrum of bat body size had slightly lower (yet still >90%) survival probability (Fig. 3).

### Species-area and species-species relationships

**Figure 4:**
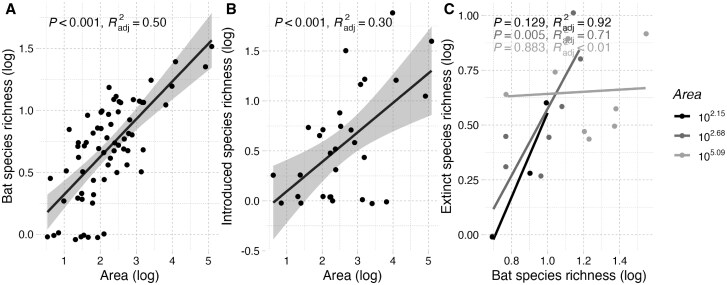
Species-area and species-species relationships. A, all (living and extinct) bat species as a function of area *S_bats_* = 1.04 × *A*^0.3^. B, introduced vertebrate species as a function of area *S_introduced_* = 0.63 × *A*^0.3^. C, extinct bat species as a function of all bat species and area log(*S_extinct_*) = −1.60 + 2.1 log(*S_bats_*) + 0.41 log(*A*) −0.39 log(*S_bats_*) log(*A*). The extinct bat species richness-area relationship was not significant (*t*_17_ = 1.689, *P *= .110).

There were strong and significant species-area relationships for all bat species and for introduced mammalian species (Fig. 4A, B); the number of introduced mammals increased with species area. The number of extinct bat species per island was not a statistically significant function of island area (*t*_17_ = 1.689, *P *= .110), indicating extinction/extirpation numbers did not increase with island size. Instead, the number of extinct species was a function of the total number of bat species interacting with island area (Fig. 4C). As a result, the number of bat extinction events depended on total species richness instead of island area.

## Discussion

We present the first comprehensive assessment of the factors underlying the resilience of bat populations in a global biodiversity and extinction hotspot (Turvey *et al.* 2017). We recovered a non-linear association of survival probability with the intrinsic trait of body mass and found that body mass interacts with two extrinsic island characteristics: area and elevation. We did not find a direct impact of island area on extirpation probability, and instead show that recent forest loss is a negative covariate of survival probability.

Here, we find that intermediate-sized bats are the least likely to persist over time in the Caribbean. Non-linear relationships between body mass and mammalian survival have been previously identified for non-volant mammals in the Caribbean (Hansford *et al.* 2012, Turvey *et al.* 2021), and also show interactions with another biotic driver of extinction, time since first human arrival. By contrast, we show that body mass interacts with environmental island features, even though those same environmental factors do not directly covary with survival probability. Neither island size nor island size change since the LGM predicted increased likelihood of bat survival across the Caribbean. Deforestation—a key ecological factor in extirpation—did co-vary with survival probability, although the relationship between recent land use change and typically older extirpation events is not straightforward (Stoetzel *et al.* 2016, Soto-Centeno *et al.* 2025). Our approach for integrating population-level data with both species traits and island features thus provides important new insights into the ecological mechanisms fostering survival in bat communities in this biodiversity and extinction hotspot.

Among non-volant mammals, the nonlinear pattern of survival arises from different threats at the high and low end of body size distributions but bats show a different pattern. Introduced mammal species have competed with or preyed on smaller-bodied endemic non-volant mammals (e.g. smaller rodents, eulipotyphlans), and human hunting targeted larger-bodied species (e.g. sloths, primates, larger rodents) (Cooke *et al.* 2017, Turvey *et al.* 2017). Thus, threats for different body size classes among different taxa of non-volant mammals are distinct with taxa *c*. 1000 g having the highest survivorship (Hansford *et al.* 2012). Conversely, while non-volant mammals were likely targeted for human consumption in the Caribbean (Cooke *et al.* 2017, Turvey *et al.* 2021), there is no evidence of past human bat consumption in this region (Mildenstein *et al.* 2016). Globally, bats face extrinsic threats from habitat degradation and loss, as well as invasive predators (Frick *et al.* 2020) that are compounded by their slow life history traits. Many bat populations, even in the tropics, only give birth to one or two pups per year, which slows recovery following mortality and puts them at greater risk of extinction (Cardillo 2003, Barclay *et al.* 2004). In the Caribbean, low reproductive rates and predation by introduced species have also been recorded for species such as the Critically Endangered Jamaican flower bat *Phyllonycteris aphylla* (Koenig and Davalos 2015), an intermediate size bat which was declared extinct twice (Oelbaum *et al.* 2024). Our analysis revealed that bat species of intermediate size (9–22 g) have a slightly lower probability of survival than both larger and smaller-bodied species, showing threats for non-volant taxa likely differ from those affecting bat populations.

Smaller body size profiles compared to non-volant mammals likely explain this finding, as Caribbean bat species are typically within the size range of small non-volant mammals disproportionately threatened by invasive species. Rates of species introductions exponentially increased after the arrival of Europeans in the Caribbean (Kemp *et al.* 2020a, b). Cats, dogs, rats, and mongooses, all of which are now present as invasive populations across many Caribbean islands, are known to prey on bat populations (Welch and Leppanen 2017). Feral cats in particular represent a severe threat to many bat populations (Welch and Leppanen 2017). Invasive rats (*Rattus rattus* and *Rattus norvegicus*), which are now ubiquitous across nearly all Caribbean terrestrial ecosystems, also prey on bats (Gloza-Rausch *et al.* 2025). Further research is needed to evaluate how invasive predators target bats of differing sizes. Size selectivity of predation risk from invasive mammals may also be associated with differing ecologies of bat species of different sizes, for instance associated with specific roosting sites and behaviours. These patterns of size selective species loss may alter bat community structure and ecological interactions therefore influencing future evolution in the Caribbean.

We propose that the pattern of size-related extinction risk found in Caribbean bats may be associated with their unique capacity for powered flight. While flight can reduce predation risk, bats of all sizes remain vulnerable while roosting. At the same time, flight enables larger bats to reduce their extinction probability, as dispersal obscures extirpation by enabling subsequent recolonization (Stoetzel *et al.* 2016). Larger-bodied bats may be more resilient to extirpation because of their dispersal abilities. Likelihood of survival in bats increases with geographic range size (Cardillo *et al.* 2008), in turn determined by dispersal ability (Chichorro *et al.* 2019) (Table 2). Several species most commonly experiencing extirpation were intermediate-sized Caribbean endemic species, including *Monophyllus redmani* (10.55 g), *Macrotus waterhousii* (12.5 g), and *Mormoops megalophylla* (15.95 g). Larger bats are capable of maintaining connectivity between islands and recolonizing following extirpation events. For example, the large-bodied species *Artibeus jamaicensis* (41.6 g) has maintained gene flow with mainland populations in Mesoamerica (Larsen *et al.* 2010), while the intermediate-sized species *Macrotus waterhousii* (12.5 g) is differentiated into multiple island endemic populations that are isolated from one another and the mainland (Fleming *et al.* 2010). Similarly, larger-bodied bat genera, such as the Caribbean endemics *Erophylla* and *Brachyphylla* appear to have largely avoided extirpation (except for *Brachyphylla* on Jamaica) through inter-island dispersal (Muscarella *et al.* 2011), with *Brachyphylla cavernarum* exhibiting relatively recent range expansions into the Lesser Antilles (Carstens *et al.* 2004, Dávalos and Turvey 2012).

### Interactions between body size and island features

Under a neutral model of biodiversity such as the equilibrium theory of island biogeography, species richness is in dynamic equilibrium between colonization and extinction (MacArthur and Wilson 1967). As island area determines extinction rates, larger islands should experience fewer extinction events relative to their species richness (MacArthur and Wilson 1967). Island isolation can also influence extinction through the rescue effect, with immigration from nearby landmasses reducing extinction risk, particularly among bats with good dispersal capability. Our results confirm that the main determinant of extirpated richness is not area, but total richness, yet larger islands show similar numbers of extirpation events regardless of their total number of bat species (Fig. 4C). Such neutral dynamics, however, assume that extinction risk is independent of intrinsic species traits and depends solely on island features. Instead, we found that island characteristics, elevation and island size, modulate body mass-dependent bat extirpation probability.

Elevation increased the probability of body mass-dependent bat survival (Fig. 3A). This result contrasts with previous findings in non-volant Caribbean mammals, which show a negative correlation between elevation and survival probability across islands, a pattern mainly driven by the many non-volant species extinctions in the high-elevation island of Hispaniola (Turvey *et al.* 2021). For bats, elevation may mitigate body-size mediated extirpation risk by increasing habitat complexity, providing refugia from storms for forest-dwelling bats and reducing risks from flooding of low-lying caves (Morgan 2001). While we found no direct effect of diet, roosting, or hurricanes on the probability of extirpation, higher elevations do offer refuge from storm damage and flooding that affects both forests and cave roosts. Our findings reveal that instead of directly influencing extirpation probability, elevation modifies body mass-dependent risk in ways consistent with offering refuge to bats of all diets and roosting habits.

Unexpectedly, island area *decreased* the body-size dependent probability of survival (Fig. 3B), with smaller islands supporting higher survival across bats of all sizes compared to larger islands. We propose three explanations, methodological and/or ecological, for this paradoxical result: extirpation observation bias towards larger islands, an increase in extirpations as a function of total richness at intermediate island size classes (Fig. 4C), and an increase in introduced species with increasing island size (Supplementary Table S8). This bias may emerge through sampling: with smaller faunas and fewer known species, and few fossil sites given their small area, smaller islands are often poorly sampled for bat fossils (Dávalos and Russell 2012), leading to fewer documented extirpations. Our analyses confirm this, as we did not observe extirpations on islands <36 km^2^, several of which only have one known bat species (bottom left, Fig. 4C). These smaller islands might not sustain permanent bat populations; persistence might only be maintained through periodic recolonization from nearby islands and our model does not account for such dynamics. By contrast, the larger islands of the Greater Antilles have experienced 33 documented extirpation events concentrated on the well-sampled islands of Cuba, Hispaniola, and Cayman Brac, comprising a major centre of bat extirpation. These extirpation events have shaped the Greater Antillean bat fauna, such that it is now in long-term disequilibrium and would require at least 8 Myr of colonization to recover its long-term species numbers (Valente *et al.* 2017). Smaller islands may also benefit from favourable metapopulation dynamics, as small islands in the Caribbean do not harbour locally endemic bat species and may therefore be rescued by immigration and recolonization from larger-island population sources. In contrast, larger-island losses often involved vulnerable island-endemic populations that could not otherwise be replaced by recolonization (e.g. *Natalus primus*, which was previously thought to be extinct) (Tejedor *et al.* 2004). Our contingency analysis of survival vs. single- or multi-island range (Table 2) supports this explanation with almost four times more extinctions of single-island endemics than extirpations of species that occur across multiple islands. Therefore, our results also highlight how the extinction of single island endemic species may have disproportionate evolutionary consequences as the loss of these lineages cannot be offset through recolonization from populations elsewhere in the Caribbean.

Larger islands could also decrease their body-size mediated probability of bat survival because they have more species than small islands, or because they have more introduced predators and herbivores. Species-area analyses revealed the number of extirpation events scales to the number of bat species for all but the largest islands. For smaller (up to ∼141 km^2^) and medium-sized islands (up to ∼479 km^2^), the probability of extirpation rises with the number of species that can become extirpated. But this explanation cannot apply to islands in the largest size class (e.g. Andros, Cuba, Guadeloupe, Jamaica, Martinique), which had roughly similar numbers of extirpation events (Fig. 4C). Instead, species introduced by humans, which follow a power scaling law in relation to island size, and their resulting ecological interactions, may explain losses on larger islands (Fig. 4B). These patterns align with the growing recognition that anthropogenic impacts, more than natural biogeographic processes, shape island biodiversity (Helmus *et al.* 2014). Introduced predators could contribute to bat extirpations by preying on roosting bats, while introduced herbivores would exacerbate anthropogenic habitat fragmentation affecting relevant habitats. Compared to past climatic change, past human activity has contributed more to Holocene bat extinction through introductions of multiple species accompanying waves of human migration, alongside disruptions from habitat fragmentation to bat roosting ecology and foraging, thereby influencing the evolution of bat populations and communities in the Caribbean (Soto-Centeno and Steadman 2015, Kemp *et al.* 2020a, b, Soto-Centeno *et al.* 2025).

Finding that island size increases the probability of body-sized-mediated bat extirpation is unexpected because larger islands generally should have lower rates of extinction. One potential explanation is that larger islands have single-island endemics that are lost more frequently than multi-island species, while smaller islands have multi-island species subject to metapopulation dynamics including rescue. This result may therefore emerge from the combination of fewer detected extinctions in the smallest islands, their greater potential for metapopulation rescue, and the presence of island endemics coupled with massive anthropogenic transformation on larger islands, including through the introduction of many more predators and herbivores.

### Deforestation and species vulnerability

We found that 21st century deforestation is associated with decreased probability of Caribbean bat survival. Forest loss directly reduces available foraging and roosting sites, and habitat fragmentation, a consequence of forest loss, heavily influences bat extinction risk (Muylaert *et al.* 2016, Frick *et al.* 2020). But while there have been 78 bat extirpations in the Caribbean, almost none of these are the direct result of recent deforestation, as most extinct bat populations are only known from Quaternary fossil remains and were already lost before the 21st century. Despite this temporal mismatch, levels of contemporary forest cover are not a significant correlate of bat extirpation, but recent forest loss is. We propose this discrepancy reflects an ecological inheritance of longer-term local deforestation and biotic reconstruction that has been more historically prevalent on some islands; islands that have experienced greater recent habitat change might be ecologically more vulnerable to past change as well (Hooghiemstra *et al.* 2018, Wells *et al.* 2018, Fitzpatrick and Giovas 2021, Elliott *et al.* 2024). Bat extirpation pre-dating European arrival has occurred on islands including Marie Galante, Antigua, and Abaco in the Bahamas (Cooke *et al.* 2017). Small-scale crop cultivation was established by pre-Columbian peoples on many Caribbean islands, and subsequent European colonization transformed island ecosystems through more extensive forest clearing and landscape alteration to accommodate larger-scale agricultural, pastoral, and plantation systems (Hooghiemstra *et al.* 2018). During the colonial period, habitat loss was extreme on some Caribbean islands. For example, loss of old-growth forest on Puerto Rico escalated from 6% in 1770 CE to > 99% by 1899 CE, and smaller islands in the Bahamas and Lesser Antilles were similarly cleared and overcultivated (Brash 1987). By the late 19th century, many islands had lost most of their original forest cover (Wells *et al.* 2018, Castilla-Beltrán *et al.* 2020).

Forest loss has therefore been a central part of human colonization of the Caribbean island system, the long-term consequences of which appear to include bat extirpations. Given this environmental history, recent Caribbean forest loss likely signals the cumulative legacy of human-altered landscapes, and thus links to the pattern of bat extirpations across islands. More accessible and fertile landscapes may make islands that experienced substantial recent forest loss prone to human incursion and disruption in the past, making these islands more vulnerable to ancient bat losses. While metrics of the state of forest cover represent a static snapshot that fails to incorporate cumulative impacts through time, metrics of the rate of deforestation may instead reveal a dynamic system of ecological shifts, with contemporary Caribbean landscapes to be viewed as a mosaic of past and ongoing human-environmental interactions.

## Conclusion

The islands of the Caribbean are hotspots of both mammalian biodiversity and extinction (Turvey 2009). We conducted the first analyses specifically focused on evaluating patterns of bat species and extinction risk in relation to intrinsic traits and extrinsic variables. We found that bats of intermediate size experienced a slightly lower probability of survival compared to both smaller-bodied and larger-bodied bats. We also found that body size interacts with the extrinsic variables of elevation and island size, further influencing the likelihood of bat extirpation. These interactions both deviate from dynamic equilibrium island biogeography expectations and support previous findings of long-term disequilibrium in Caribbean bats caused by anthropogenic extinction events (Valente *et al.* 2017). Finally, deforestation co-varies with likelihood of bat extirpation, probably reflecting the ecological inheritance of habitat loss and anthropogenic disturbance across the Caribbean islands. These findings highlight the importance of body mass and its interactions with extrinsic factors in shaping extinction patterns, with broader implications for island biogeography, macroecology, and the conservation of insular bat faunas.

## Supplementary Material

kzag014_Supplementary_Data

## Data Availability

The data and code underlying this article are available in the DRYAD digital repository (Rayfield *et al*. 2026), at https://doi.org/10.5061/dryad.hhmgqnkwn.
