# Supplementary material for: Interactions between intrinsic and extrinsic factors shape island bat survival and extirpation in a global extinction hotspot
Source: Evol J Linn Soc. 2026 Jun 15;5(1):kzag014. doi: 10.1093/evolinnean/kzag014 (PMC13387144; doi:10.1093/evolinnean/kzag014)
Supplement: kzag014_Supplementary_Data [file kzag014_supplementary_data.zip › Suppl.pdf]

Table S10. Phylogenetic regression estimates of the mean and standard deviation (sd) of imputed body mass for species lacking direct observations based on one phylogeny from (Upham et al. 2019) and the alternate phylogeny of (Alvarez-Carretero et al. 2021). All but the *Lasiurus* and *Sturnira* species are extinct. No greatest length of skull was available for *Mormoops magna* or *Pteronotus* sp. nov.; results are from phylogenetic imputation.

Species	mean	sd	mean (alternate)	sd (alternate)
Artibeus anthonyi	40.73	1.24	41.04	1.28
Cubanycteris silvai	32.58	1.21	31.43	1.25
Desmodus puntajudensis	15.42	1.47	15.67	1.51
Lasiurus degelidus	11.90	1.33	10.79	1.43
Lasiurus minor	8.95	1.33	8.82	1.28
Mormoops magna	11.83	1.73	8.84	1.68
Phyllonycteris major	24.38	1.17	24.04	1.27
Phyllops silvai	19.74	1.12	20.94	1.11
Phyllops vetus	14.80	1.12	15.96	1.11
Pteronotus pristinus	10.14	1.38	10.53	1.44
Pteronotus sp. nov.	9.73	1.82	9.31	1.8
Sturnira paulsoni	19.55	1.17	19.18	1.34

For Review Only

Table S11. Summary of model coefficients for the three models based on one phylogeny from (Upham et al. 2019) and the alternate phylogeny of (Alvarez-Carretero et al. 2021). Statistically supported variables are in **bold**. Lower = lower 95% credible interval, upper = upper 95% credible interval.

	No imputed body mass			Imputed body mass			Only significant covariates			No imputed body mass (alternate phylogeny)			Imputed body mass (alternate phylogeny)			Only significant covariates (alternate phylogeny)		
Covariate	mean	lower	upper	mean	lower	upper	mean	lower	upper	mean	lower	upper	mean	lower	upper	mean	lower	upper
intercept	**4.56**	**1.38**	**7.94**	3.33	-0.54	7.38	2.75	-0.34	6.44	**4.41**	**1.49**	**7.76**	**3.42**	**0.18**	**6.87**	**2.75**	**0.30**	**5.55**
body mass	0.46	-0.96	1.94	0.49	-1.08	1.98	0.81	-0.67	2.21	0.53	-0.92	2.06	0.48	-1.08	1.98	0.82	-0.58	2.17
body mass^2	**1.09**	**0.24**	**2.06**	**0.88**	**0.04**	**1.84**	**0.82**	**0.04**	**1.72**	**1.12**	**0.24**	**2.17**	**0.92**	**0.05**	**1.90**	**0.84**	**0.04**	**1.72**
diet	**3.06**	**0.28**	**6.58**	2.71	-0.67	7.86				3.03	-0.07	6.93	2.71	-0.76	7.54			
cave	-3.82	-6.79	-0.87	-1.22	-3.83	1.76				-3.60	-6.54	-0.71	-1.45	-4.11	1.41			
area	0.07	-0.99	0.87	-0.18	-1.21	0.57	-0.37	-1.18	0.22	0.07	-0.98	0.85	-0.18	-1.23	0.56	-0.38	-1.20	0.20
area change	-0.17	-1.15	0.85	-0.19	-1.11	0.79				-0.18	-1.15	0.82	-0.19	-1.13	0.80			
elevation	0.08	-0.97	1.18	0.19	-0.81	1.23	0.58	-0.12	1.37	0.11	-0.93	1.19	0.19	-0.83	1.24	0.59	-0.12	1.38
hurricane frequency	-0.22	-1.18	0.69	-0.25	-1.15	0.60				-0.21	-1.14	0.69	-0.25	-1.17	0.62			
first human arrival	-0.38	-1.64	0.65	-0.60	-1.81	0.36				-0.36	-1.57	0.66	-0.61	-1.83	0.36			
proportion forest cover 2000	0.56	-0.27	1.51	0.69	-0.09	1.61				0.57	-0.26	1.50	0.70	-0.09	1.64			
proportion forest loss to 2000	**-1.00**	**-1.79**	**-0.40**	**-0.92**	**-1.68**	**-0.35**	**-0.74**	**-1.30**	**-0.31**	**-1.00**	**-1.78**	**-0.40**	**-0.93**	**-1.70**	**-0.35**	**-0.74**	**-1.31**	**-0.31**

For Review Only

	No imputed body mass			Imputed body mass			Only significant covariates			No imputed body mass (alternate phylogeny)			Imputed body mass (alternate phylogeny)			Only significant covariates (alternate phylogeny)		
Covariate	mean	lower	upper	mean	lower	upper	mean	lower	upper	mean	lower	upper	mean	lower	upper	mean	lower	upper
intercept	**4.56**	**1.38**	**7.94**	3.33	-0.54	7.38	2.75	-0.34	6.44	**4.41**	**1.49**	**7.76**	**3.42**	**0.18**	**6.87**	**2.75**	**0.30**	**5.55**
body mass	0.46	-0.96	1.94	0.49	-1.08	1.98	0.81	-0.67	2.21	0.53	-0.92	2.06	0.48	-1.08	1.98	0.82	-0.58	2.17
body mass^2	**1.09**	**0.24**	**2.06**	**0.88**	**0.04**	**1.84**	**0.82**	**0.04**	**1.72**	**1.12**	**0.24**	**2.17**	**0.92**	**0.05**	**1.90**	**0.84**	**0.04**	**1.72**
diet	**3.06**	**0.28**	**6.58**	2.71	-0.67	7.86				3.03	-0.07	6.93	2.71	-0.76	7.54			
cave	-3.82	-6.79	-0.87	-1.22	-3.83	1.76				-3.60	-6.54	-0.71	-1.45	-4.11	1.41			
area	0.07	-0.99	0.87	-0.18	-1.21	0.57	-0.37	-1.18	0.22	0.07	-0.98	0.85	-0.18	-1.23	0.56	-0.38	-1.20	0.20
mean human footprint index	0.74	-0.22	1.86	0.68	-0.20	1.75				0.74	-0.21	1.83	0.70	-0.19	1.79			
Body mass by area	**-0.61**	**-1.21**	**-0.06**	**-0.76**	**-1.28**	**-0.29**	**-0.70**	**-1.18**	**-0.25**	**-0.59**	**-1.19**	**-0.05**	**-0.79**	**-1.32**	**-0.32**	**-0.72**	**-1.21**	**-0.27**
Body mass^2 by elevation	**0.90**	**0.28**	**1.57**	**0.78**	**0.22**	**1.38**	**0.70**	**0.19**	**1.27**	**0.89**	**0.27**	**1.56**	**0.78**	**0.22**	**1.38**	**0.69**	**0.18**	**1.24**

For Review Only

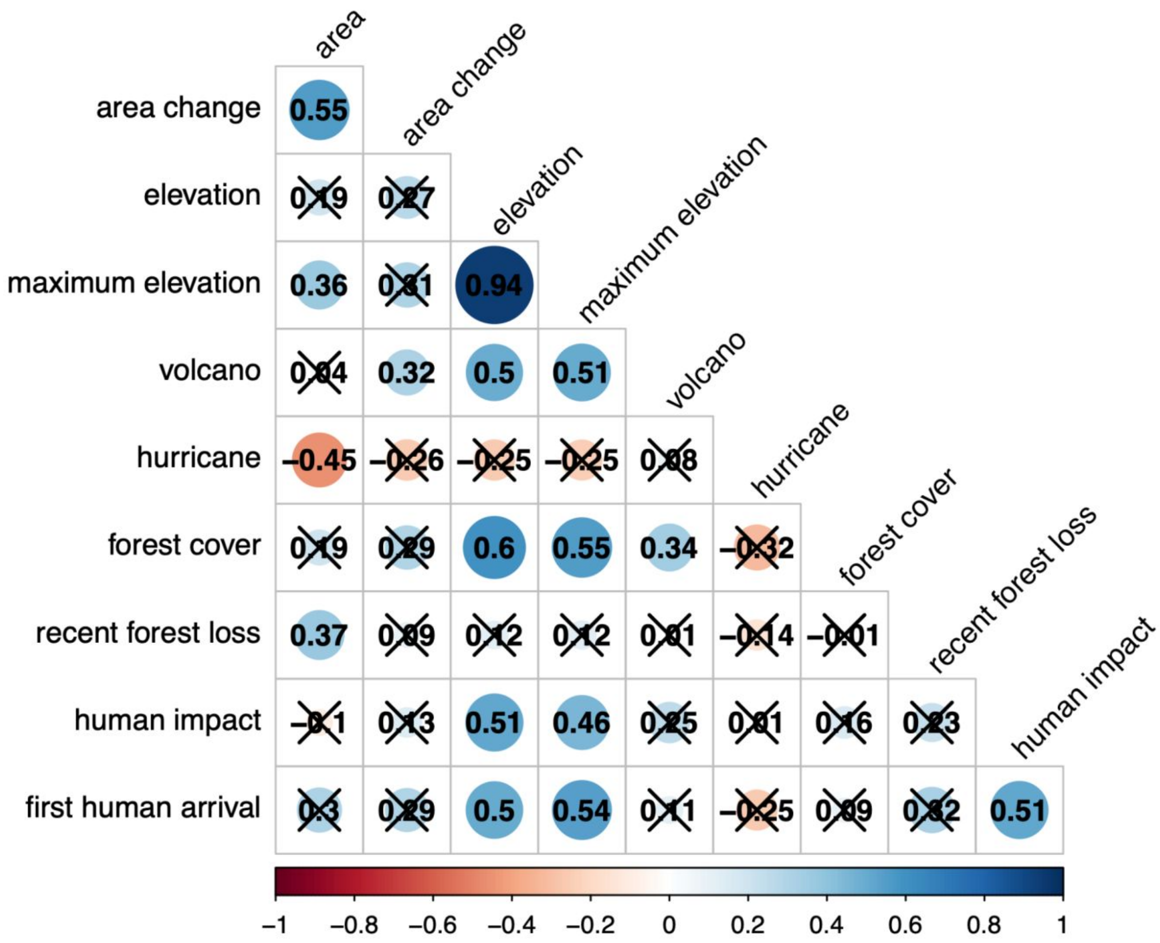

Figure S1. Bivariate Pearson correlations showing calculated *R*. Positive correlations are in blue, negative in red, and non significant values are crossed out.

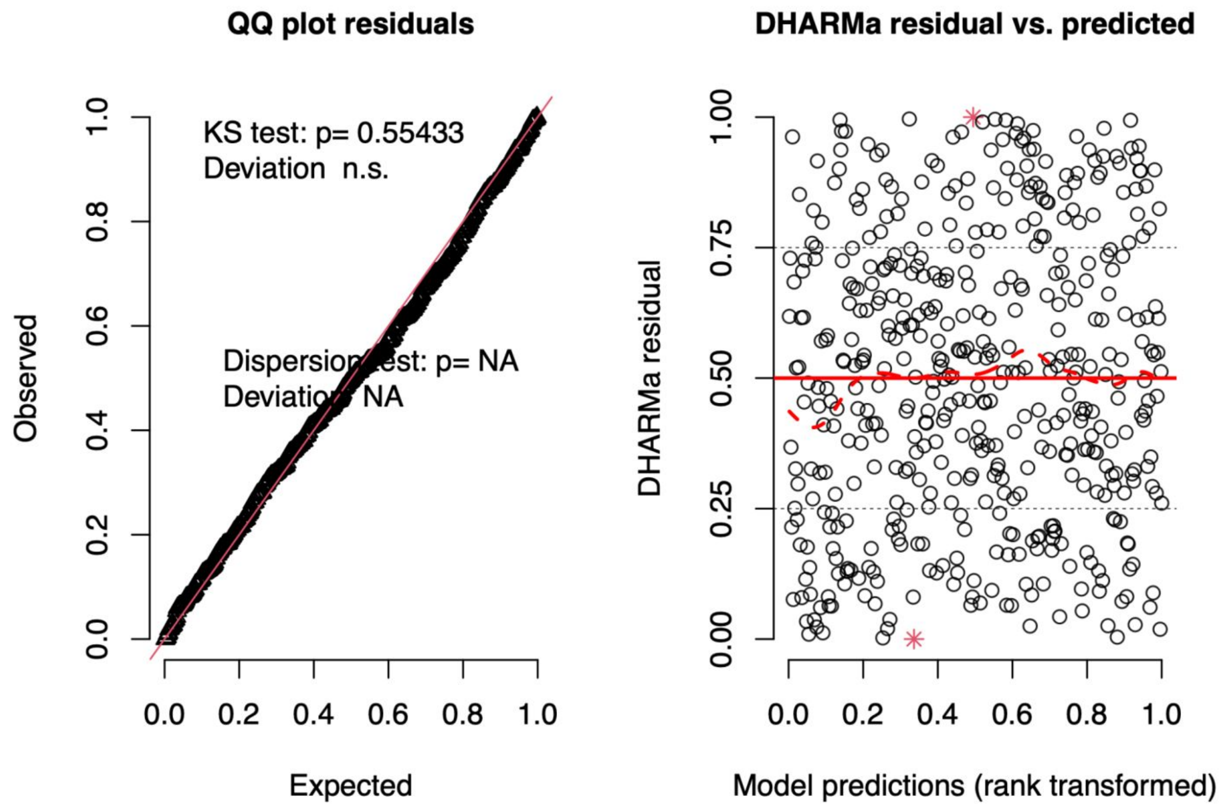

Figure S2. Model assumption checks through analysis of binomial regression residuals simulated using DHARMa. Residuals show no significant deviation or dispersion, while two residuals indicate potential outliers.

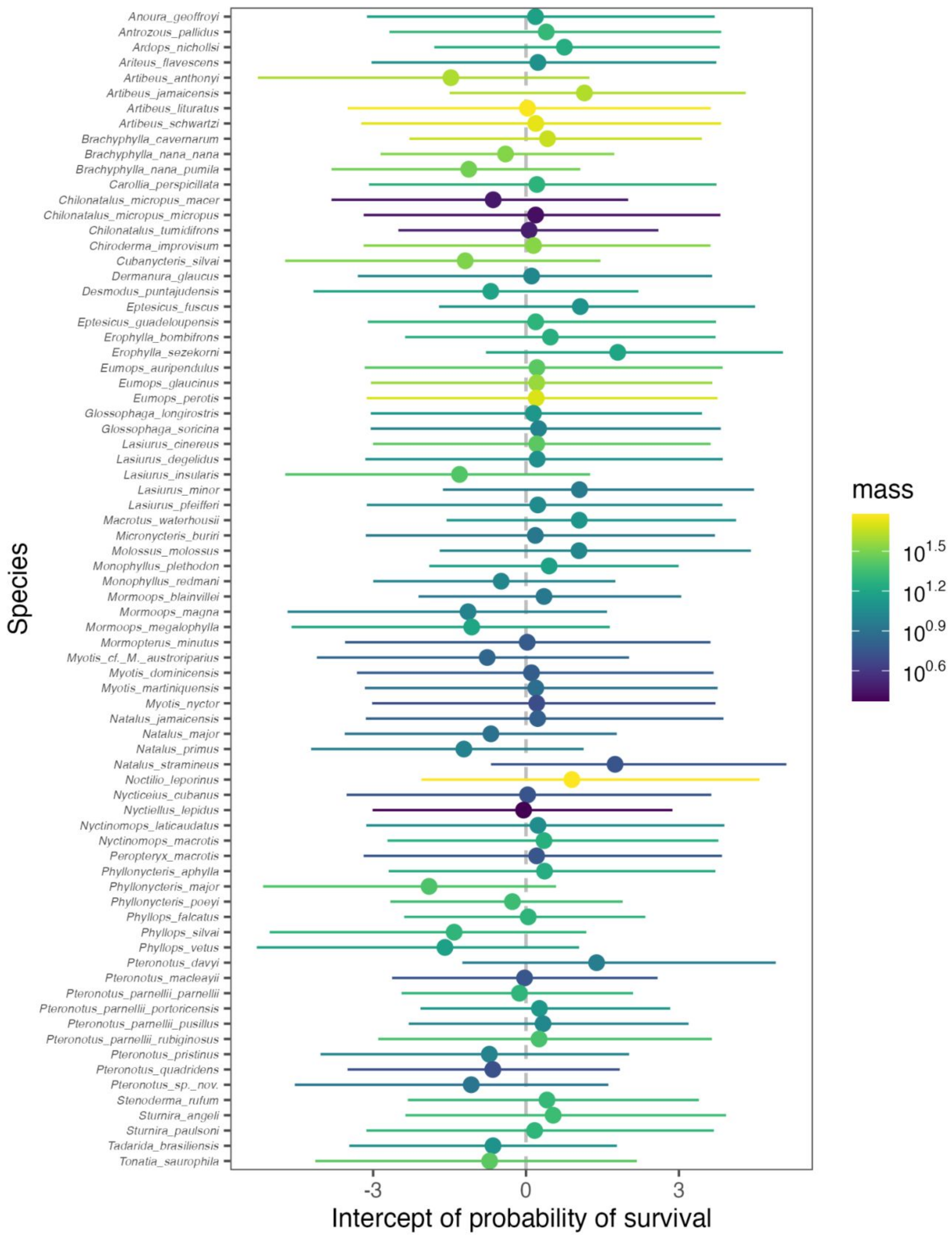

Figure S3. Species-specific non-phylogenetic coefficients on the probability of bat population survival. All effects have credible intervals overlapping 0 and are therefore not statistically supported.

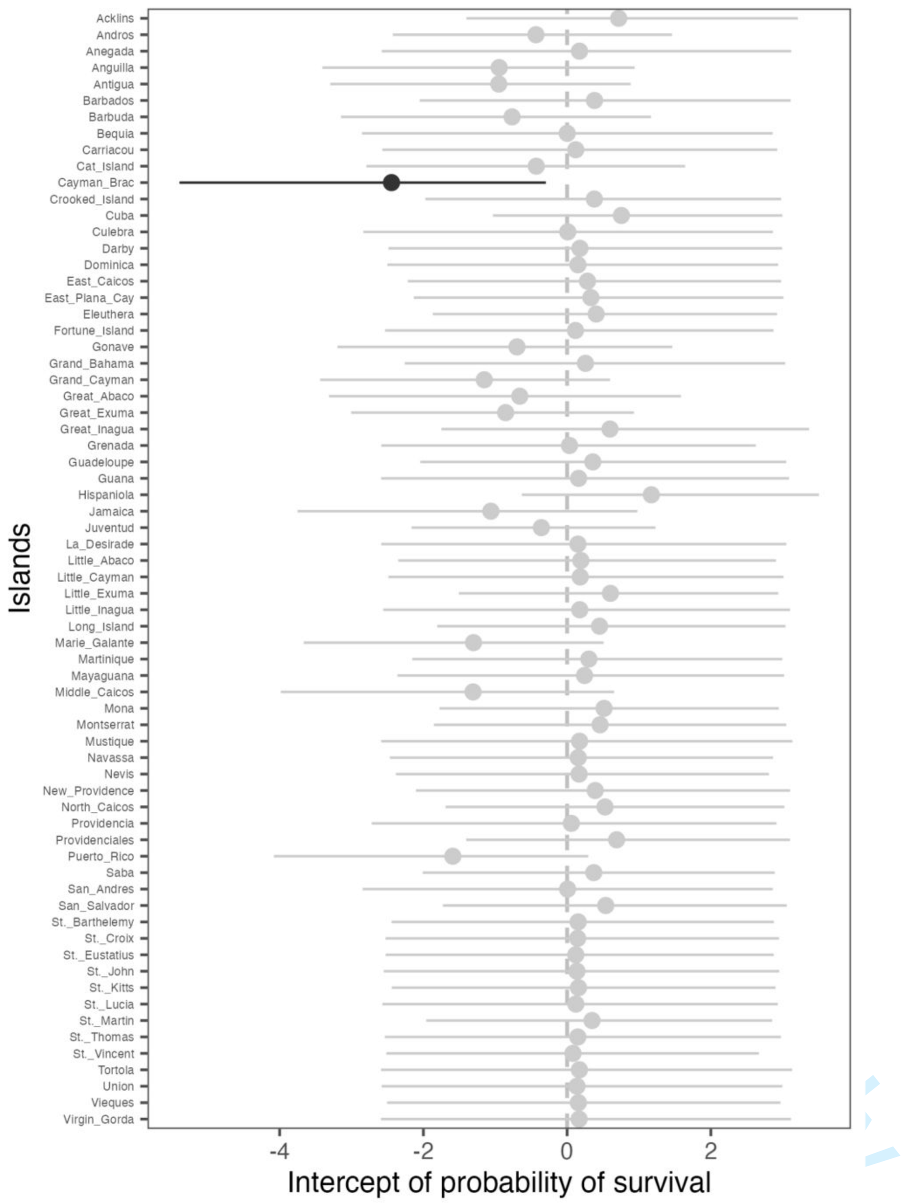

Figure S4. Island-specific coefficients on the probability of bat population survival. The effect credible interval lower than 0 is shown in black.
